# *Ficus pumila L.* improves the prognosis of patients infected with HTLV-1, an RNA virus

**DOI:** 10.1186/s12937-021-00672-x

**Published:** 2021-02-11

**Authors:** Kenji Gonda, Koichi Suzuki, Yumi Sunabe, Koji Kono, Seiichi Takenoshita

**Affiliations:** 1 Daido Central Hospital, 1-1-37 Asato, Naha, Okinawa, 902-0067 Japan; 2 Fukushima Medical University, 1 Hikarigaoka, Fukushima, 960-1295 Japan

**Keywords:** *Ficus pumila L.*, HTLV-1, miRNA, Ooitabi

## Abstract

Human T-cell leukemia virus type 1 was isolated as the retrovirus to be identified in humans. Here, we focused on *Ficus pumila L.* as a factor that be effective against human T-cell leukemia virus type 1. The significant and novel findings is that symptoms of patients with drinking *Ficus pumila L.* extracts did not worsen despite a lack of aggressive pharmacotherapy against adult T-cell leukemia, a human T-cell leukemia virus type 1-associated myelopathy, or T-cell leukemia virus type 1 uveitis. Twenty-eight of the 194 inpatients who underwent showed high levels of human T-cell leukemia virus type 1.

Among human T-cell leukemia virus type 1-infected patients, those who were administered *Ficus pumila L*. extracts had no human T-cell leukemia virus type 1-related symptoms, while those who were not administered *Ficus pumila L.* extracts had human T-cell leukemia virus type 1-related diseases and a significantly poorer prognosis. This suggests that the *Ficus pumila L.* extracts may show some utility against virus infection.

## Main text

Human T-cell leukemia virus type 1 (HTLV-1) has been isolated from humans. HTLV-1 is a type C retrovirus that is primarily endemic to Japan, Central and South America, the Middle East, regions of Africa, and the Caribbean. HTLV-1 and related diseases are particularly common in southwestern Japan, especially in rural areas of Kyushu and Okinawa, and Japan is the only developed nation that is an HTLV-1-endemic country.

HTLV-1 was first isolated and described by Montagnier and Gallo, and is the first pathogenic retrovirus to be identified in humans. HTLV-1 is a retrovirus that causes malignant tumors of cluster of differentiation 4 (CD4)-positive T lymphocytes, namely adult T-cell leukemia (ATL), along with other diseases such as HTLV-1-associated myelopathy (HAM), an intractable neurological disorder, and HTLV-1 uveitis (HU). ATL tends to be more common in men, and HAM and HU tend to be more common in women, and the virus is mainly transmitted from men to women. Given that women are particularly susceptible to HTLV-1 infection, carrier mothers are forced to wean their infants shortly after childbirth, causing anxiety and mental distress.

**Fig. 1:**
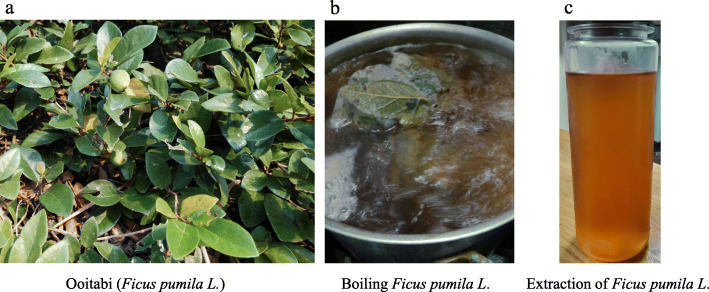
**a**. An Ooitabi (*Ficus pumila L.*) plant winds up on stone walls. **b**. Placing 100 g of clean washed *Ficus pumila L*. leaves in 1 L of boiling water at 100 °C for 10 min produces a brown liquid. **c**. Extraction of *Ficus pumila L*. is called ‘Ishimaki tea’, which is mixed with a flavor and heated for about 2 min to yield the more palatable Ishimaki tea, compared with the original extract

Numerous flavonoid-rich plants are distributed in Okinawa. One such plant, Ooitabi (*Ficus pumila L.*), inhabits warm areas, such as Okinawa, where is found in rocky areas (Fig. 1. a). An infusion obtained by boiling Ooitabi (Fig. 1. b) is called “Ishimaki tea” (Fig. 1. c), and it has been used by the islanders as a folk remedy for diabetes and hypertension since ancient times [1].

**Fig. 2:**
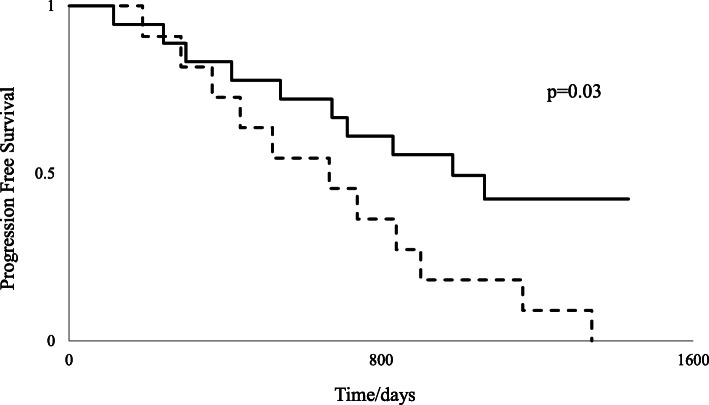
The prognosis of HTLV-1-positive patients. Patients administered Ooitabi (*Ficus pumila L.*) (solid line) had a significantly better prognosis than those who were not (dashed line). A crossing of the Progression Free Survival curves was noted, representing a delay in benefit with inhibitors that may be typical with immunotherapy, but survival improves and eventually Tail plateaus over time. A similar prognosis was observed with immune checkpoint inhibitors. *Ficus pumila L.* may restore the original function of T cells and suppress tumor activation. The HTLV-1 antibody was measured by the gelatin particle agglutination method (BML, Inc., Japan). When measuring Progression Free Survival, follow-up data that were reached as of the last follow-up date or at 1500 days after treatment onset were used. Patient prognoses were analyzed using the Kaplan Meier method and the log rank test was used to determine the univariate significance of the variables. *P* < 0.05 was considered to indicate statistical significance

Twenty-eight of the 194 inpatients who underwent HTLV-1 testing from January 2016 to December 2020 were HTLV-1-positive. Nineteen were female (median 86.4 age (58–97 years old)) and nine were male (median 88.1 age (82–94 years old)). Of these, 12 inpatients developed symptoms (42.8%); 9 were women and 3 were men. Specifically, 8 were HAM, 2 were ATL, and 2 were HU. HTLV-1-infected cells undergo up-down regulation of microRNA (miRNA) [2]. *Ficus pumila L.* contains flavonoids such as rutin and apigenin [3], which have been shown to reduce the expression of miR-155 and miR-146a and increase the expression of miR-31. The expression of miR-155 was increased in HTLV-1-positive T-cell lines [4]. Overexpression of miR-146a enhances the growth of HTLV-1-infected T-cell lines [5]. miR-31 expression is relatively high in normal T cells, but very low in ATL cells [6]. Here, we describe the application of *Ficus pumila L*. against HTLV-1. Among HTLV1-infected patients, those who were administered *Ficus pumila L*. extracts had no HTLV-1-related symptoms, while those who were not administered *Ficus pumila L*. extracts had HTLV-1-related diseases and a significantly poorer prognosis (Fig. 2). The administered *Ficus pumila L*. extracts contained approximately 2.441 mg/200–300 g leaves/day of rutin and approximately 1.411 mg/200–300 g leaves/day of apigenin. In another study, pretreatment with troxerutin had the effect of decreasing expression levels of miR-146a and miR-155 in a diabetic group as compared to the control [7]. Apigenin- and apigenin-rich diets exert effective anti-inflammatory activity in vivo by reducing LPS (lipopolysaccharide)-induced expression of miR-155, thereby restoring immunity [8]. *Ficus pumila L.* also contains kaempferol. The expression levels of miR-31, KRAS oncogene, and the c-MYC transcription factor were subexpressed upon 72 h post-treatment with kaempferol-3-O-glycoside compared with the control without Treatment [9]. miR-146a was down-regulated by kaempferol treatment [10].

Although no significant difference was observed between treatments, drinking *Ficus pumila L*. extracts decreased soluble interleukin 2 receptor levels, which is a marker of lymphoma, and improved lower limb muscle weakness and gait disorder due to slowly progressive bilateral leg spasm paresis. The ability to walk was restored and urinary tract infections, pollakiuria, and constipation due to bladder and rectal disorders all improved.

Since coronavirus is a single-stranded, plus strand RNA virus and the genome itself acts as mRNA, it is presumed that up-down regulation by miRNA in the early stage of infection is effective for therapeutic miRNA. Ingestion of flavonoid-rich *Ficus pumila L*. may inhibit viral transcription and replication, and therapeutic agents for HTLV-1 may be effective against coronavirus 2019-nCoV infection. Although HTLV-1 is zoonoses - the origin of a part of African HTLV-1 was monkeys, it was able to infect human cells due to mutations resulting from repeated RNA rearrangements. RNA is a key component of all multicellular life, and the fact that it is involved in animal and human transmission is thus unsurprising. If *Ficus pumila L*. can suppress the replication of the HTLV-1 retrovirus, and if HTLV-1 therapeutics work against single-stranded RNA virus infections, then *Ficus pumila L*. may be an effective therapeutic agent against 2019-nCoV retroviral infection. This suggests that the *Ficus pumila L*. extracts may show some utility against coronavirus 2019-nCoV infection.

### Limitations

Due to the small number of subjects, this study cannot show a cause–effect relationship strictly. Social acceptance and recall bias were also possible confounding factors.

## Data Availability

The dataset supporting the conclusion of this article is available from the authors on request.

## Abbreviations

HTLV-1: Human T-cell leukemia virus type 1
CD4: Cluster of differentiation 4
ATL: Adult T-cell leukemia
HAM: HTLV-1-associated myelopathy
HU: HTLV-1 uveitis
miRNAs: microRNAs
LPS: Lipopolysaccharide

## References

[1] Suzuki K, Gonda K, Kishimoto Y, Katsumoto Y, Takenoshita S. Potential curing and beneficial effects of Ooitabi (*Ficus pumila L*.) on hypertension and dyslipidaemia in Okinawa. J Hum Nutr Diet. 2020; Online ahead of print. 10.1111/jhn.12806 PMC8048928 32845065

[2] Sampey GC, Van Duyne R, Currer R, Das R, Narayanan A, Kashanchi F (2012). Complex role of microRNAs in HTLV-1 infections. Front Genet.

[3] Liao CR, Kao CP, Peng WH, Chang YS, Lai SC, Ho YL. Analgesic and Anti-Inflammatory Activities of Methanol Extract of *Ficus pumila* L in Mice. Evid Based Complement Alternat Med. 2012:340141. 10.1155/2012/340141 PMC3359828 22666289

[4] Tomita M. Important Roles of Cellular MicroRNA miR-155 in Leukemogenesis by Human T-Cell Leukemia Virus Type 1 Infection. ISRN Microbiol. 2012; Print 2012. 10.5402/2012/978607 PMC3671690 23762762

[5] Tomita M, Tanaka Y, Mori N (2012). MicroRNA miR-146a is induced by HTLV-1 tax and increases the growth of HTLV-1-infected T-cells. Int J Cancer.

[6] Yamagishi M, Nakano K, Miyake A, Yamochi T, Kagami Y, Tsutsumi A (2012). Polycomb-mediated loss of miR-31 activates NIK-dependent NF-κB pathway in adult T cell leukemia and other cancers. Cancer Cell.

[7] Yavari R, Badalzadeh R, Alipour MR, Tabatabaei SM (2016). Modulation of hippocampal gene expression of microRNA-146a/microRNA-155-nuclear factor-kappa B inflammatory signaling by troxerutin in healthy and diabetic rats. Indian J Pharmacol.

[8] Arango D, Diosa-Toro M, Rojas-Hernandez LS, Cooperstone JL, Schwartz SJ, Mo X (2015). Dietary apigenin reduces LPS-induced expression of miR-155 restoring immune balance during inflammation. Mol Nutr Food Res.

[9] Gutierrez-Uribe JA, Salinas-Santander M, Serna-Guerrero D, Serna-Saldivar SRO, Rivas-Estilla AM, Rios-Ibarra CP (2020). Inhibition of miR31 and miR92a as oncological biomarkers in RKO Colon Cancer cells treated with Kaempferol-3-O-glycoside isolated from black bean. J Med Food.

[10] Jiang R, Hao P, Yu G, Liu C, Yu C, Huang Y (2019). Kaempferol protects chondrogenic ATDC5 cells against inflammatory injury triggered by lipopolysaccharide through down-regulating miR-146a. Int Immunopharmacol.

